# Supplementary material for: A structural and functional analysis of opal stop codon translational readthrough during Chikungunya virus replication
Source: J Gen Virol. Author manuscript; Available in PMC 2024 Mar 5. (PMC7615711; doi:10.1099/jgv.0.001909)
Supplement: Supplementary material 1 [file EMS194309-supplement-Supplementary_material_1.pdf]

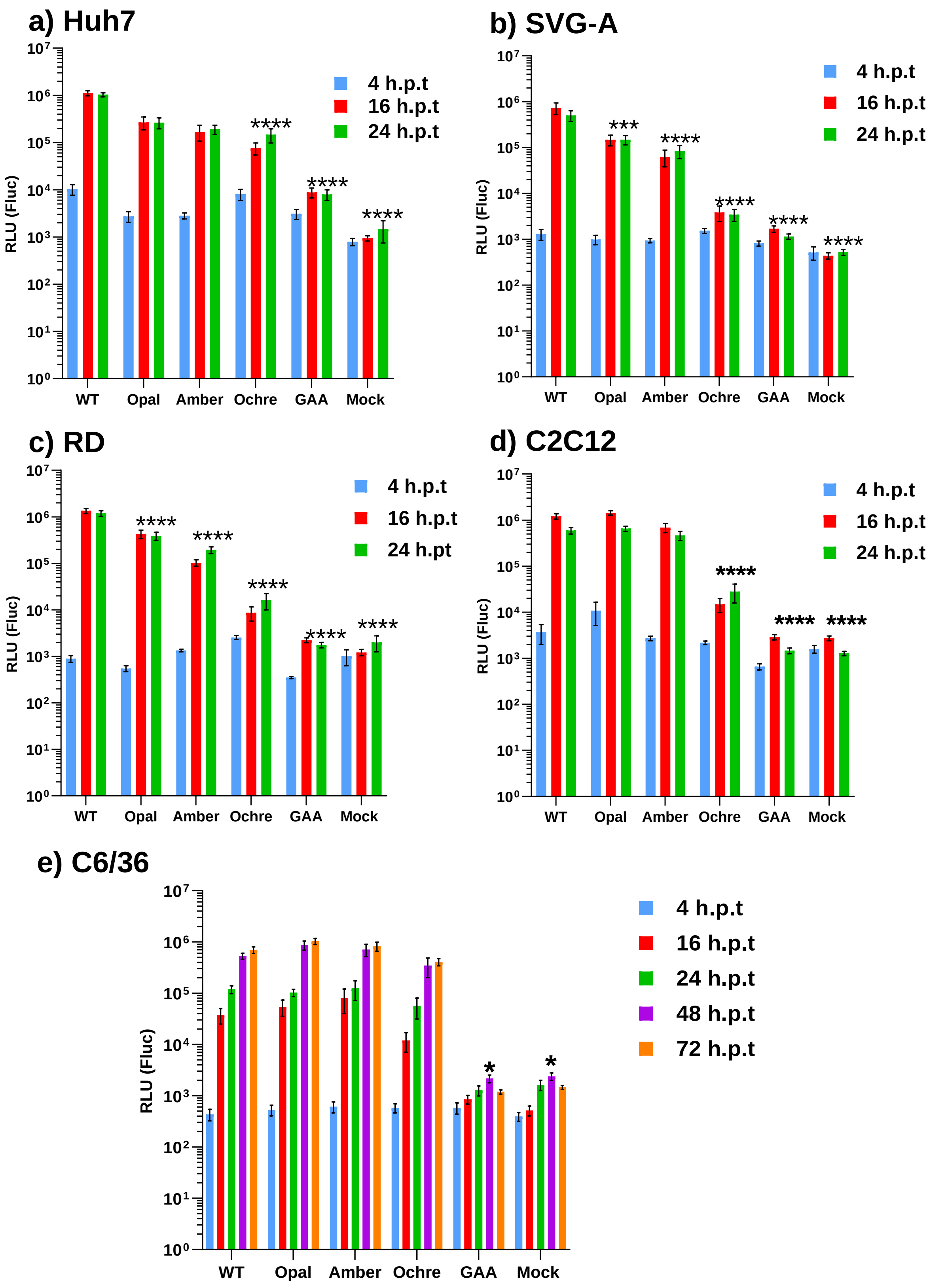

Supplementary Figure S1. Replication of CHIKV-Dluc-SGR wildtype and stop codon mutants in different cell lines. Graphs show absolute F-luc values

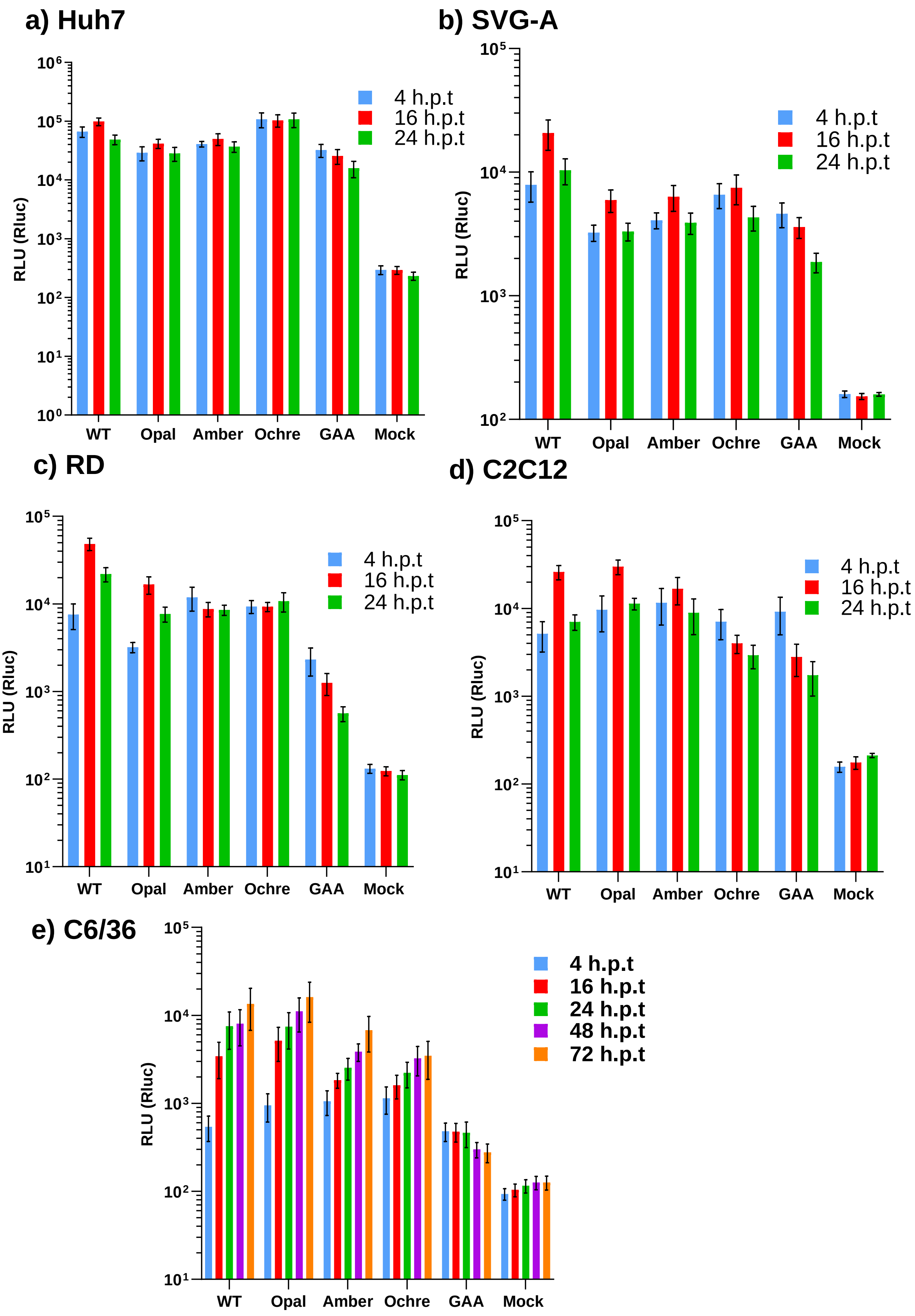

Supplementary Figure S2. Replication of CHIKV-Dluc-SGR wildtype and stop codon mutants in different cell lines. Graphs show absolute R-luc values

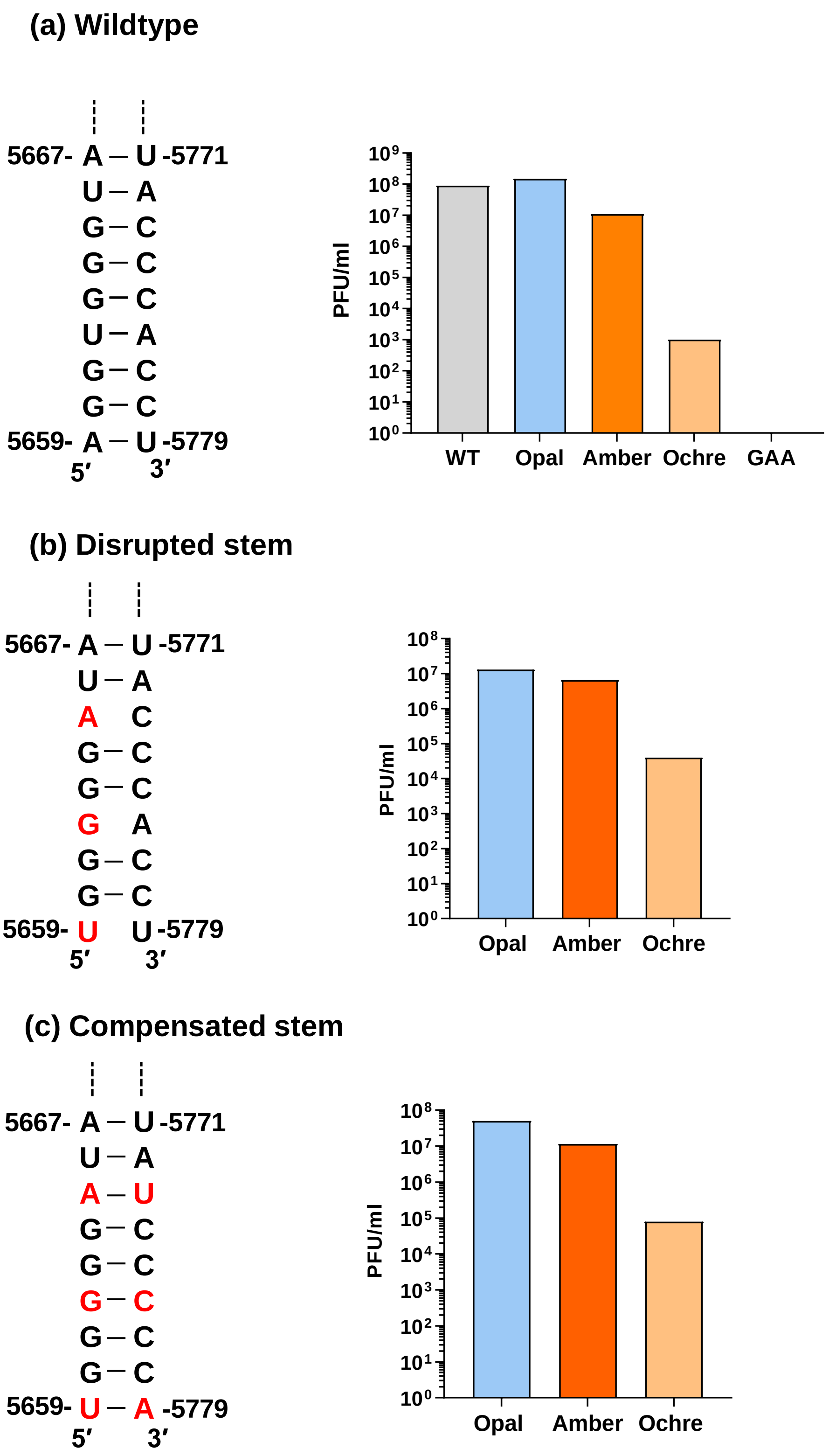

Supplementary Figure S3. Plaque assay on BHK-21 cells of virus released from electroporated C6/36 cells at 48 h, expressed as plaque forming units (PFU) per ml of clarified supernatant (n=1).
