# Supplementary material for: A structural and functional analysis of opal stop codon translational readthrough during Chikungunya virus replication
Source: J Gen Virol. Author manuscript; Available in PMC 2024 Mar 5. (PMC7615711; doi:10.1099/jgv.0.001909)
Supplement: Supplementary material 2 [file EMS194309-supplement-Supplementary_material_2.pdf]

Base position	37°C	28°C
5644	0.8775	0
5645	1.2075	0.86
5646	0.7675	0.135
5647	0.33	0.37
5648	0.675	0
5649	0.82	0.05
5650	0.6525	0.245
5651	0.4175	0.13
5652	0.2025	0.025
5653	0.1175	0.045
5654	0.505	0
5655	0.3925	0.17
5656	0.18	0
5657	0.28	0
5658	0	0
5659	0.185	0
5660	0.04	0.005
5661	0.005	0
5662	0	0
5663	0.0625	0
5664	0.05	0.45
5665	0.05	0.52
5666	0.185	1.21
5667	0.5525	0.64
5668	0.57	1.385
5669	0.9125	0.785
5670	0.6325	0.92
5671	0.7475	0.29
5672	0.3	0.325
5673	0.1625	0
5674	0.0075	0.105
5675	0.03	0
5676	0.0825	0.44
5677	0.095	0
5678	0.385	0.28
5679	0.2775	0.115
5680	0.2025	0.115
5681	0.1125	0.065
5682	0.085	0
5683	0.2125	0.06
5684	0.0525	0.1
5685	0.0425	0.075
5686	0.2	0.065

5687	0.88	0.05
5688	0.49	0.385
5689	0.1	0.095
5690	0.0025	0
5691	0.15	0.23
5692	0.285	0.44
5693	0.1175	0.215
5694	0.015	0.01
5695	0.055	0.025
5696	0.2525	0.145
5697	0.3375	0.565
5698	0.4475	0.585
5699	0.63	0.58
5700	0.625	0.75
5701	0.2175	0.09
5702	0.31	0.24
5703	0.685	0.625
5704	0.5475	0.185
5705	0.1875	0.095
5706	1.1675	0.805
5707	1.345	0.57
5708	1.39	1.095
5709	0.8625	1.525
5710	0.3275	0.045
5711	0.17	0.08
5712	0	0
5713	0.1325	0.245
5714	0.1475	0.135
5715	0.3375	0.51
5716	0.3675	0.51
5717	0.2475	0.22
5718	0.2	0.2
5719	0.115	0.02
5720	0.0075	0
5721	0.5125	0.17
5722	0.49	0
5723	0.6475	0.24
5724	0.2	0.025
5725	0.9175	0.66
5726	0.9325	1.23
5727	0.5625	0.6
5728	0.21	0.15
5729	0.0175	0.025
5730	0.0275	0
5731	0.055	0

5732	0.0875	0
5733	0.175	0
5734	0.4	0.09
5735	0.3775	0.285
5736	0.56	0.105
5737	0.8925	0.79
5738	0.2775	0.42
5739	0.2925	0.17
5740	0	0.085
5741	0.0975	0.115
5742	0.07	0.005
5743	0.035	0
5744	0	0
5745	0.0025	0
5746	0.025	0.08
5747	0	0.03
5748	0.035	0.015
5749	0.11	0.05
5750	0.4075	0.255
5751	0.7075	0.455
5752	0.74	0.385
5753	0.1325	0.09
5754	0.045	0
5755	0.01	0
5756	0	0.14
5757	0.17	0.1
5758	0.025	0.01
5759	0.03	0.02
5760	0.0925	0.125
5761	0.715	1.44
5762	0.05	0.115
5763	0.125	0.1
5764	0.3925	0.17
5765	0.6425	0.525
5766	0.6425	1.26
5767	0.995	0.195
5768	0.66	0.475
5769	0.56	0.46
5770	0.4125	0.68
5771	0.2025	0.465
5772	0	0.04
5773	0	0.025
5774	0.01	0.095
5775	0.02	0.205
5776	0.02	0.08

5777	0.0475	0.04
5778	0.0325	0.25
5779	0.2675	0.7
5780	0.65	0.865
5781	0.6875	0.66
5782	0.29	0.265
5783	0.075	0.08
5784	0.0675	0.225
5785	0.03	0.23
5786	0.0925	0.2
5787	0.205	0.335
5788	0.36	0.405
5789	0.3675	0.475
5790	0.2925	0.615
5791	0.31	0.37
5792	0.146667	0.5
5793	0.255	0.24
5794	0.195	0.73

Supplementary Table S1. Normalized SHAPE reactivities for CHIKV nucleotides 5644 to 5799 from full-length genomic RNA folded at either 37°C or 28°C, n=3.
